# Supplementary material for: ProbeST: a custom probe design pipeline for dual host–pathogen Spatial Transcriptomics
Source: BMC Genomics. 2026 Jun 25;27:561. doi: 10.1186/s12864-026-13077-z (PMC13295851; doi:10.1186/s12864-026-13077-z)
Supplement: Supplementary file 2 — Supplementary Material 2. [file 12864_2026_13077_MOESM2_ESM.docx]

**Additional file 6**


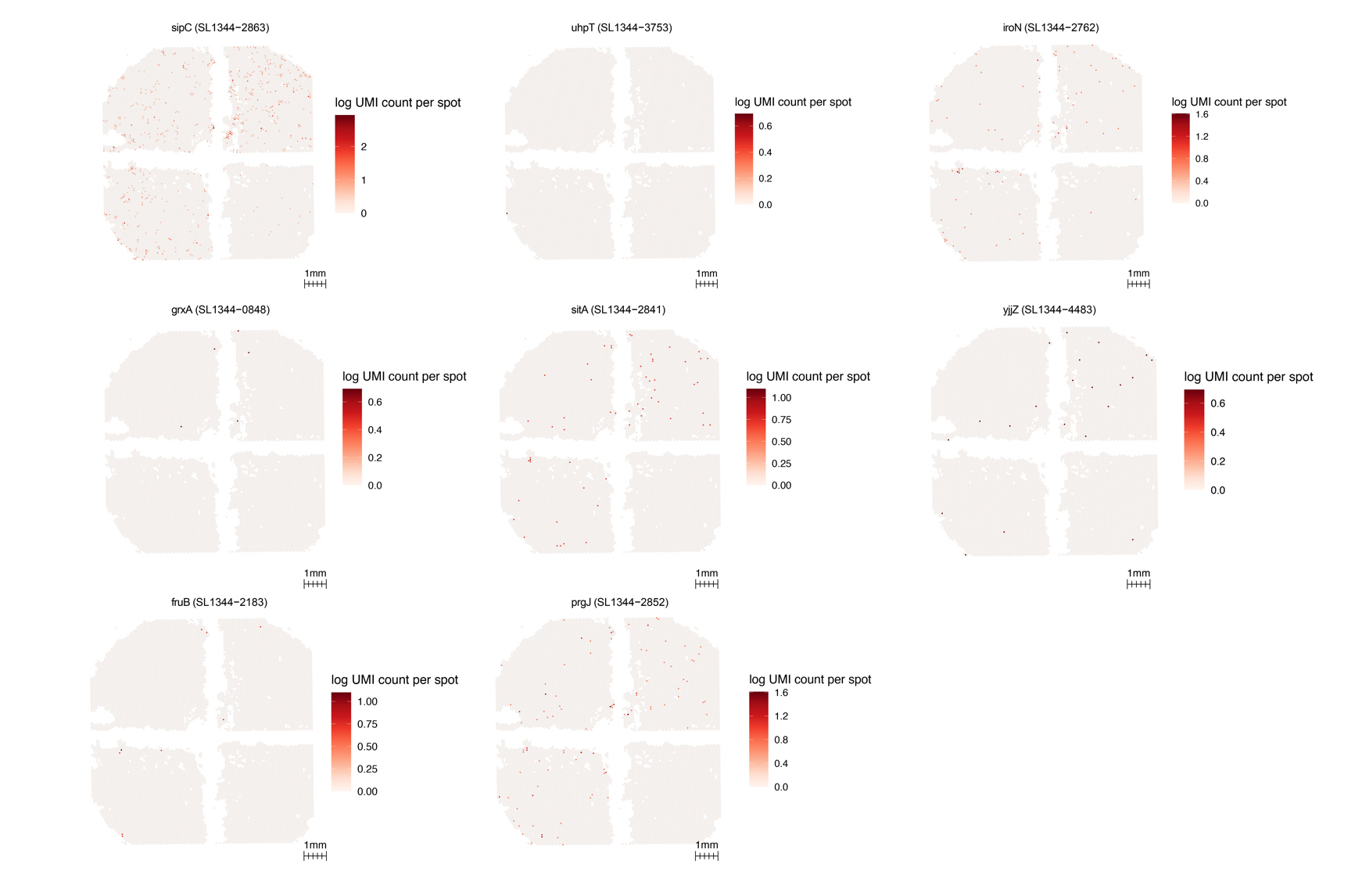


**Figure S1: Spatial *S*. Tm cytosolic gene distributions across all four sample conditions.** For each gene: top left monolayer corresponds to *GsdmD*^-/-^ infected condition, top right to *Nlrc4*^-/-^ infected condition, bottom left to WT infected condition, and bottom right to the uninfected WT condition. The UMI counts are in log1p.


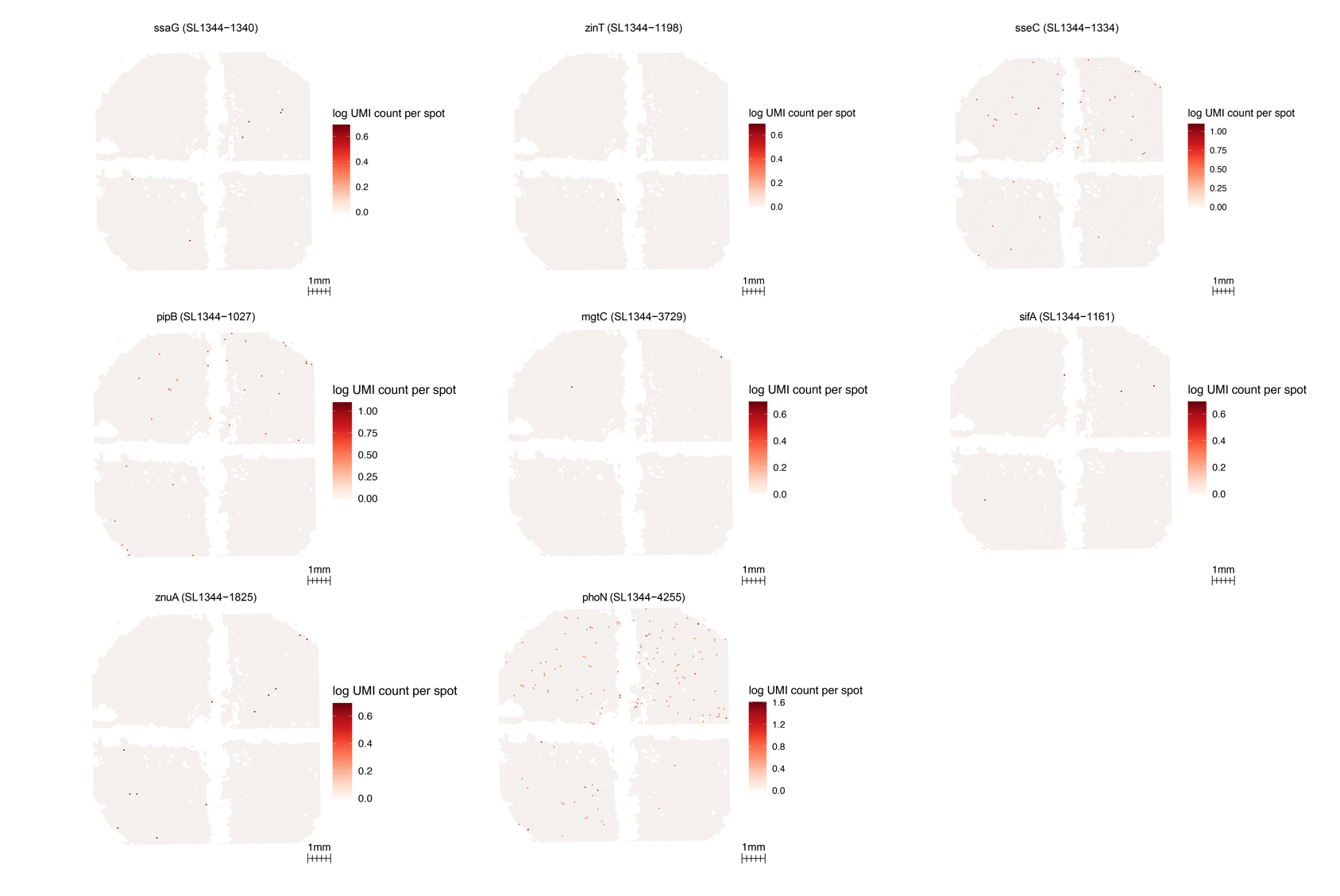


**Figure S2: Spatial *S*. Tm vacuolar gene distributions across all four sample conditions.** For each gene: top left monolayer corresponds to *GsdmD*^-/-^ infected condition, top right to *Nlrc4*^-/-^ infected condition, bottom left to WT infected condition, and bottom right to the uninfected WT condition. The UMI counts are in log1p.


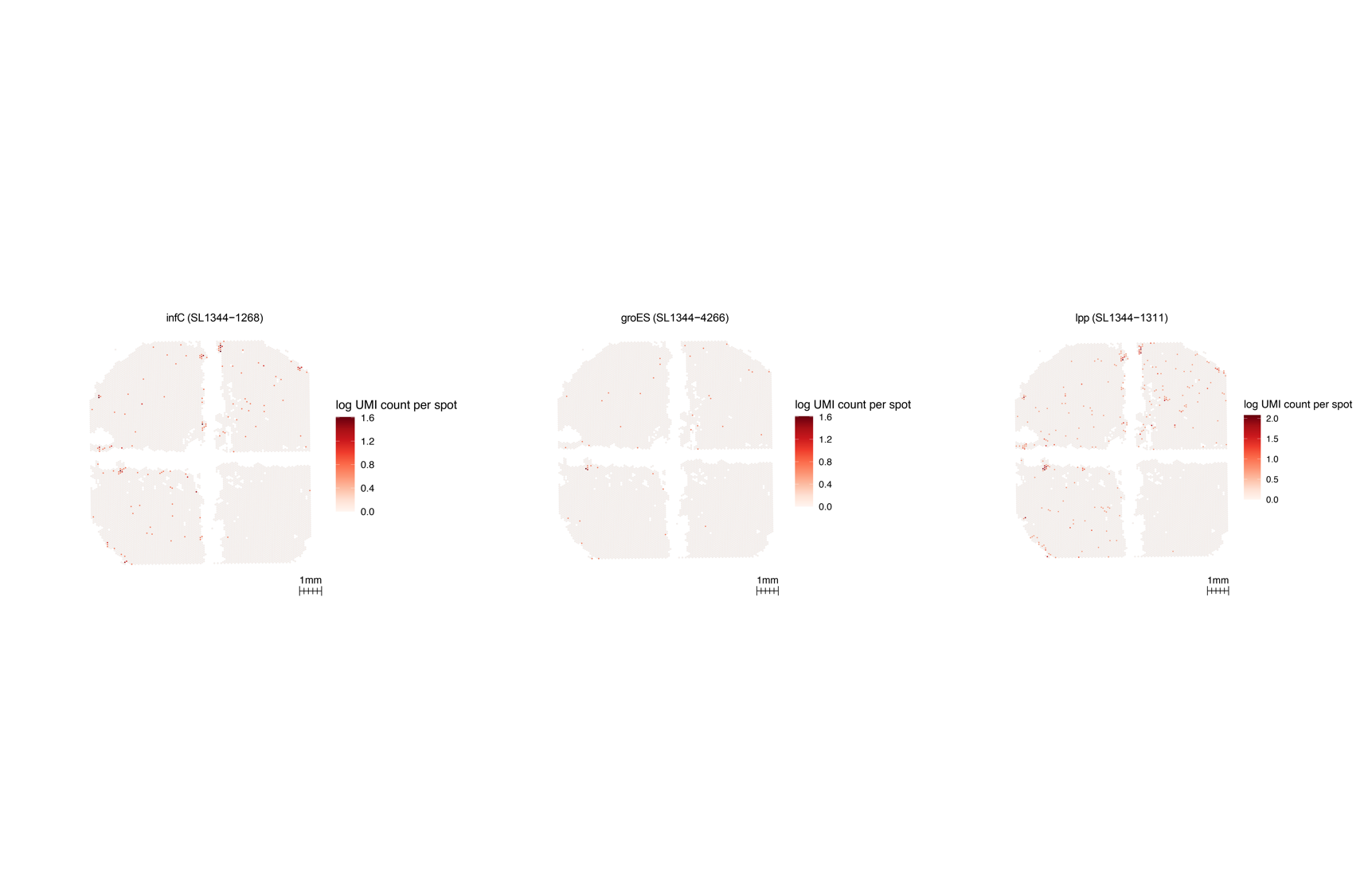


**Figure S3: Spatial *S*. Tm housekeeping gene distributions across all four sample conditions.** For each gene: top left monolayer corresponds to *GsdmD*^-/-^ infected condition, top right to *Nlrc4*^-/-^ infected condition, bottom left to WT infected condition, and bottom right to the uninfected WT condition. The UMI counts are in log1p.


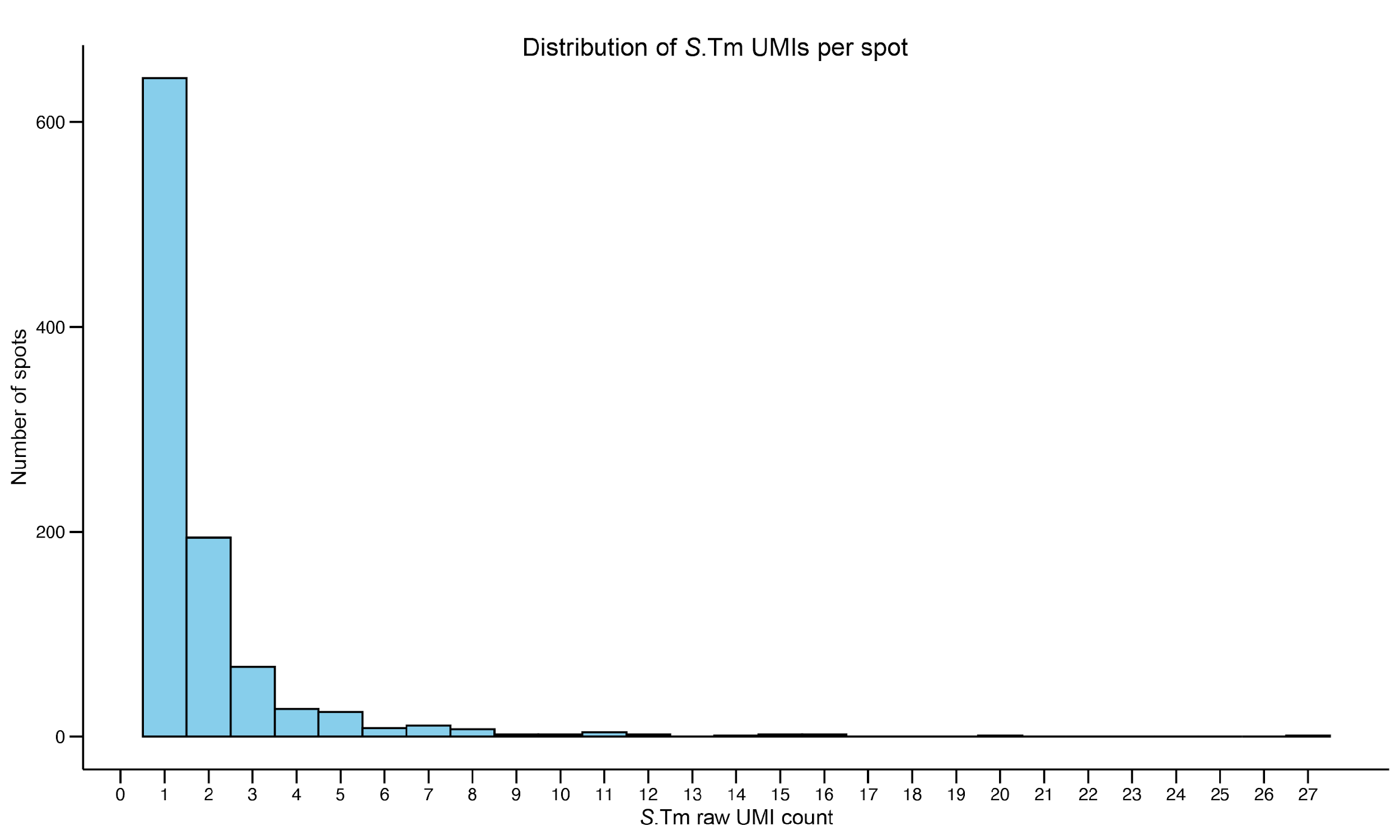


**Figure S4: Distribution of *S*. Tm UMI abundance per spot.**


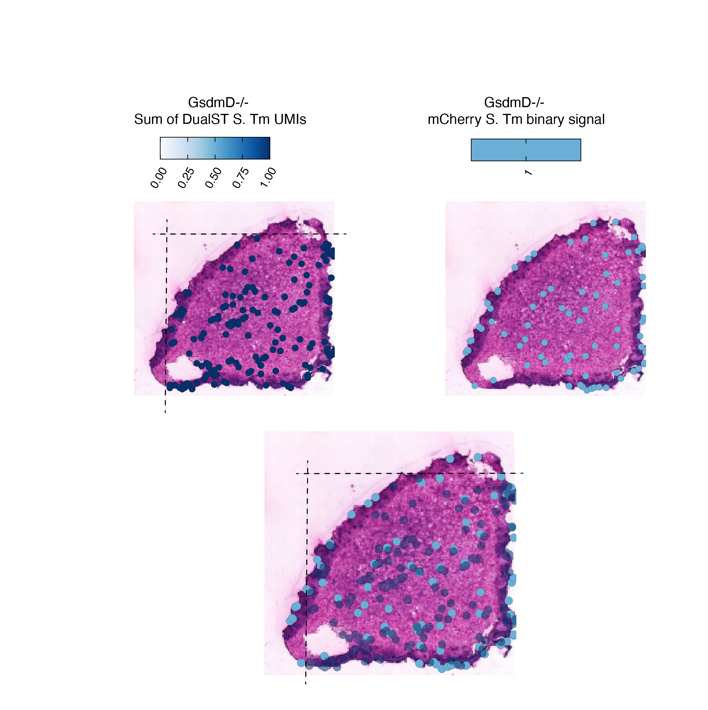

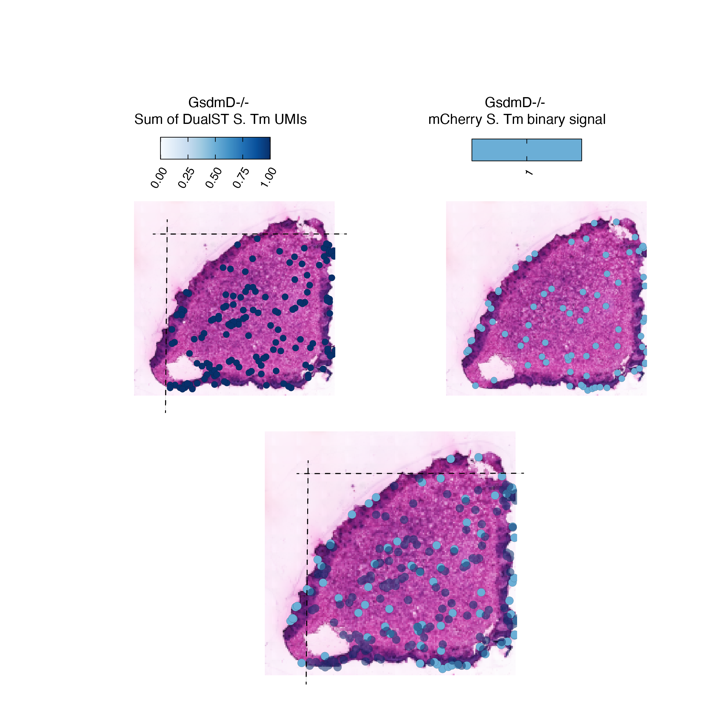


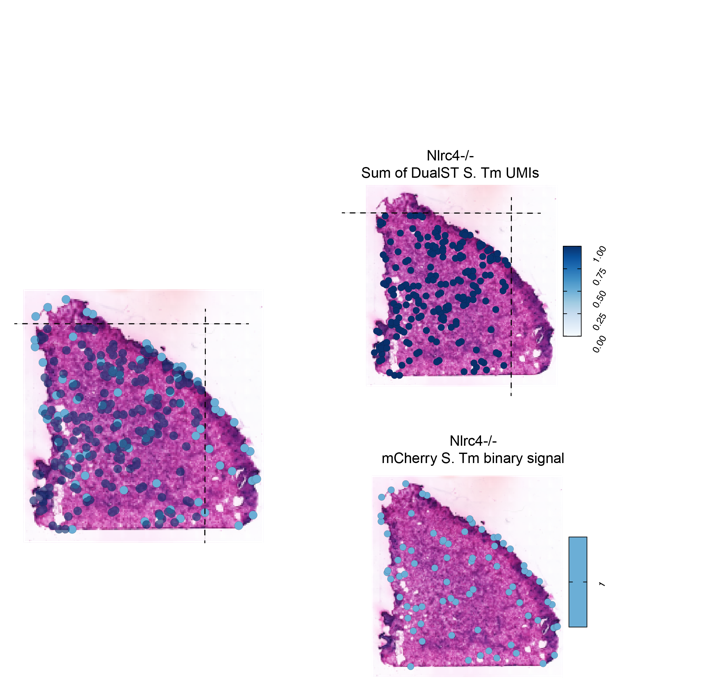

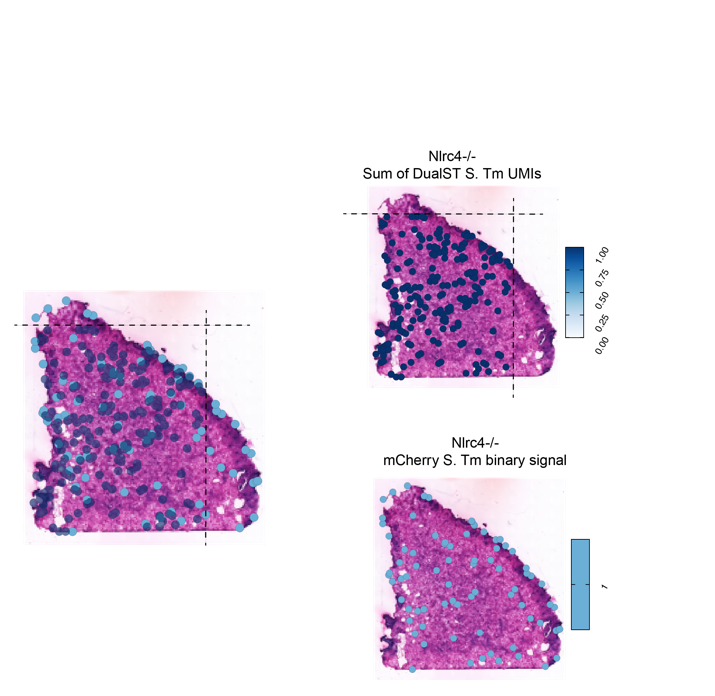

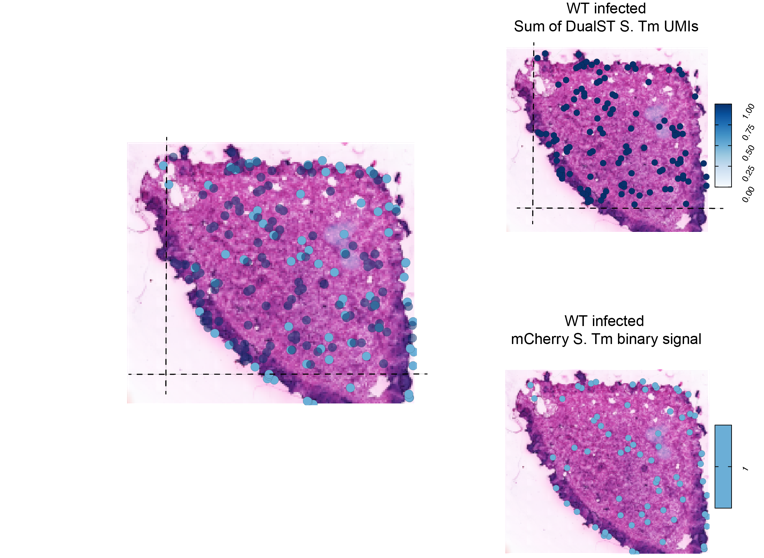

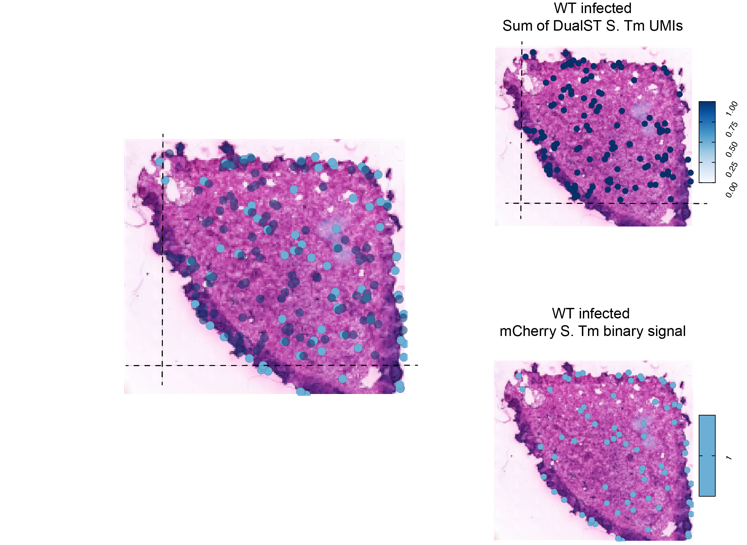


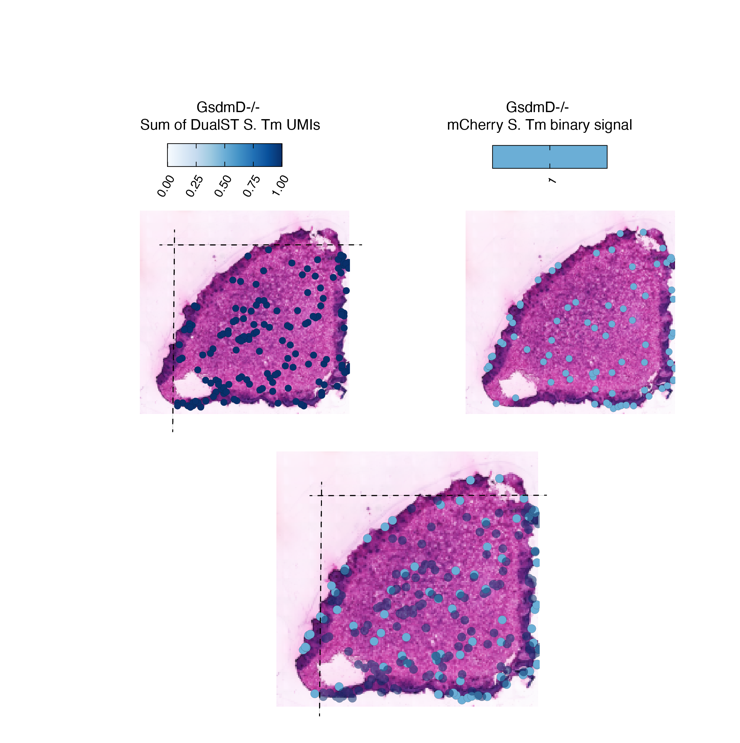

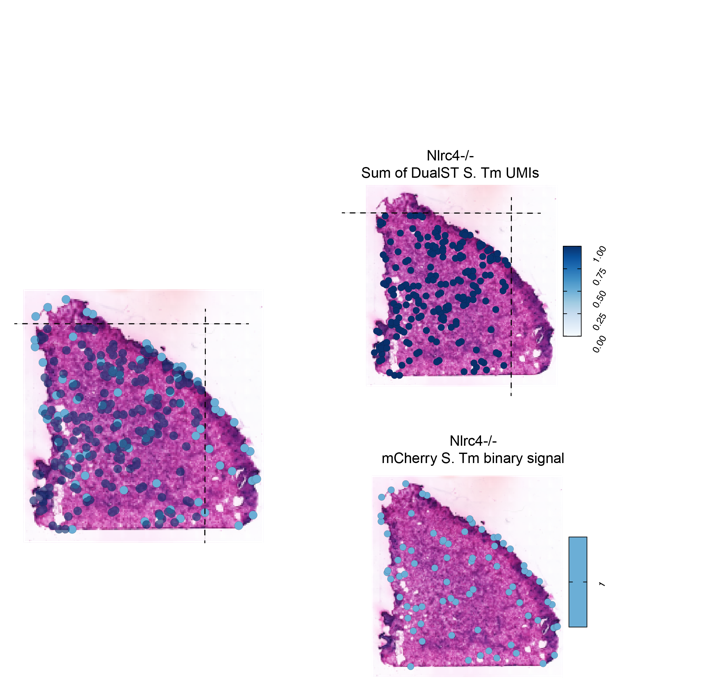

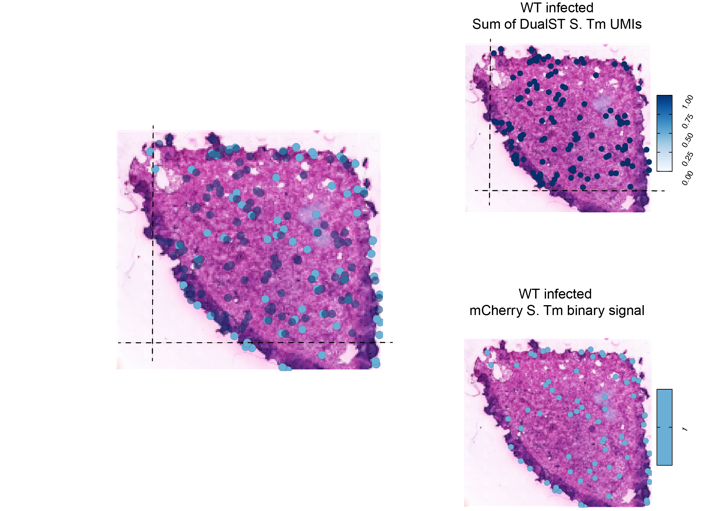


**Figure S5: DualST *S*. Tm UMIs per spot compared to the mCherry signal.** From left to right: *GsdmD*^-/-^ infected condition, *Nlrc4*^-/-^ infected condition, and WT infected condition. A 0.99 percentile cutoff was applied to the DualST *S*. Tm UMIs for better comparison to the binary mCherry signal. On the top row is presented both signals separately, for each condition. On the bottom row is the overlay of the signals on the enteroid-derived monolayers. Dashed lines represent the boundaries of the area captured by the Visium technology.


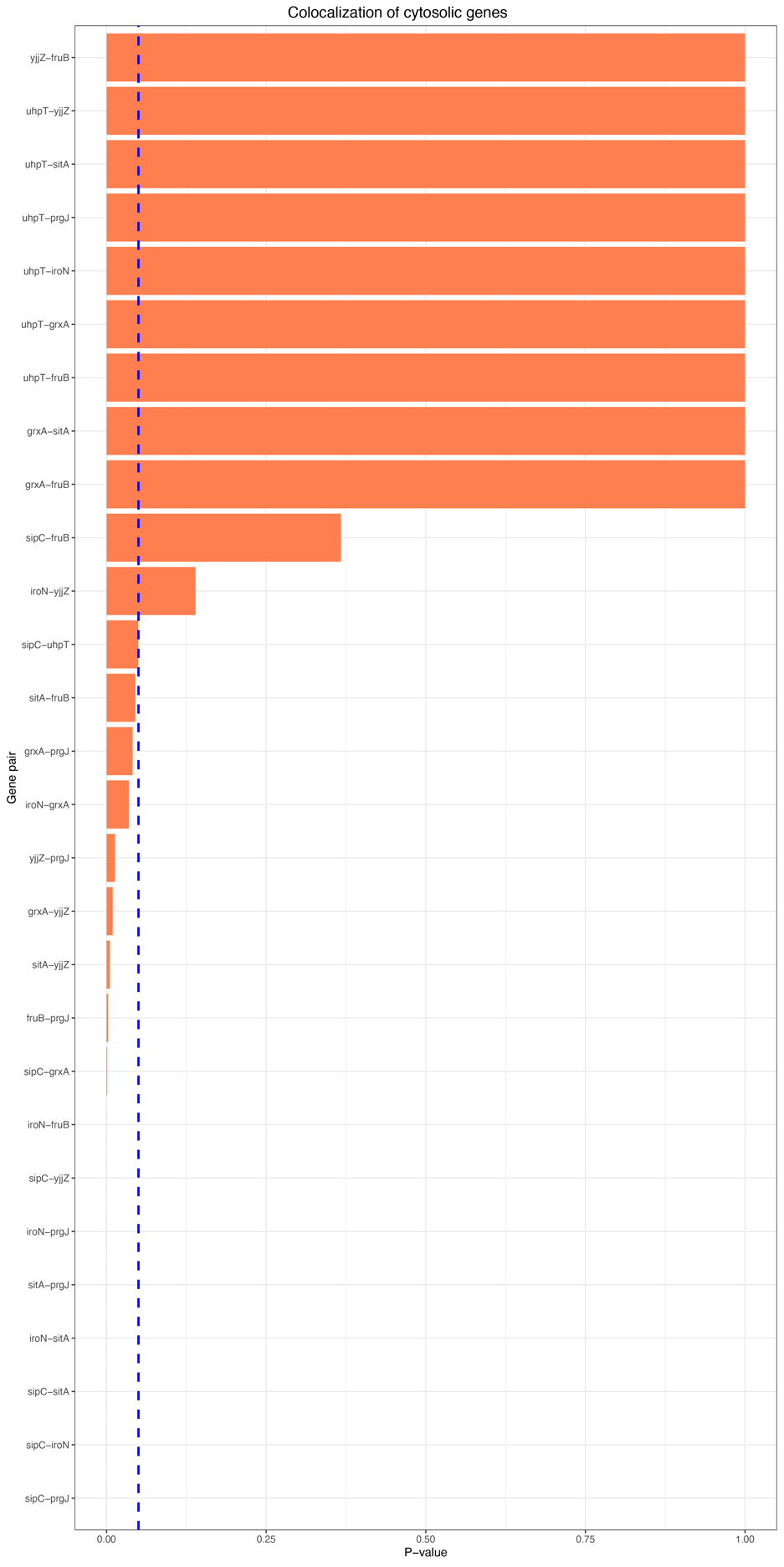


**Figure S6: *S*. Tm cytosolic gene colocalization results.** Analytical p-values for the colocalization of *S*. Tm cytosolic gene pairs across the Salmo+ spots from Fisher’s exact test. The dotted vertical line indicates a significance threshold p-value of 0.05.


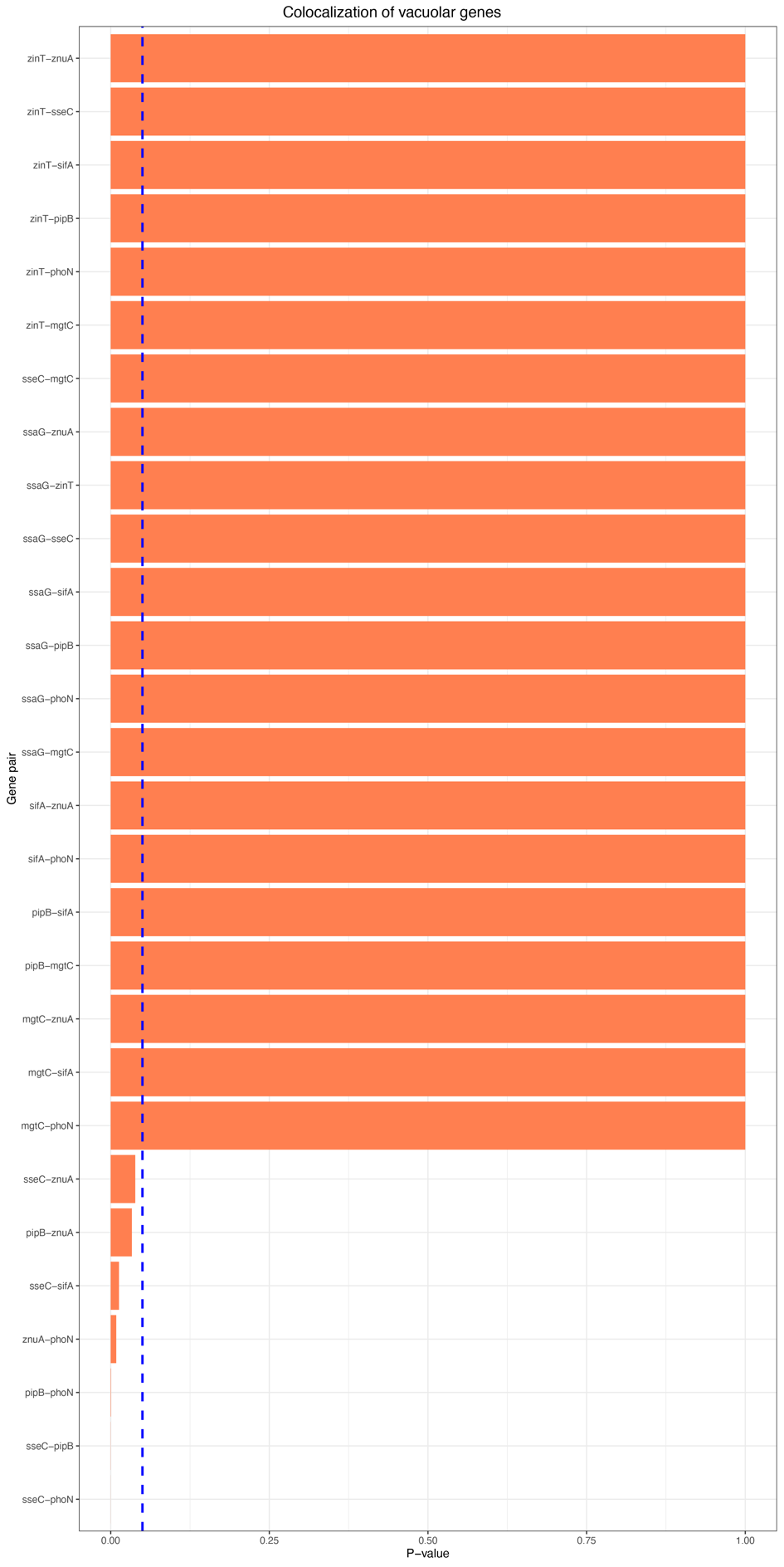


**Figure S7: *S*. Tm vacuolar gene colocalization results.** Analytical p-values for the colocalization of *S*. Tm vacuolar gene pairs across the Salmo+ spots from Fisher’s exact test. The dotted vertical line indicates a significance threshold p-value of 0.05.


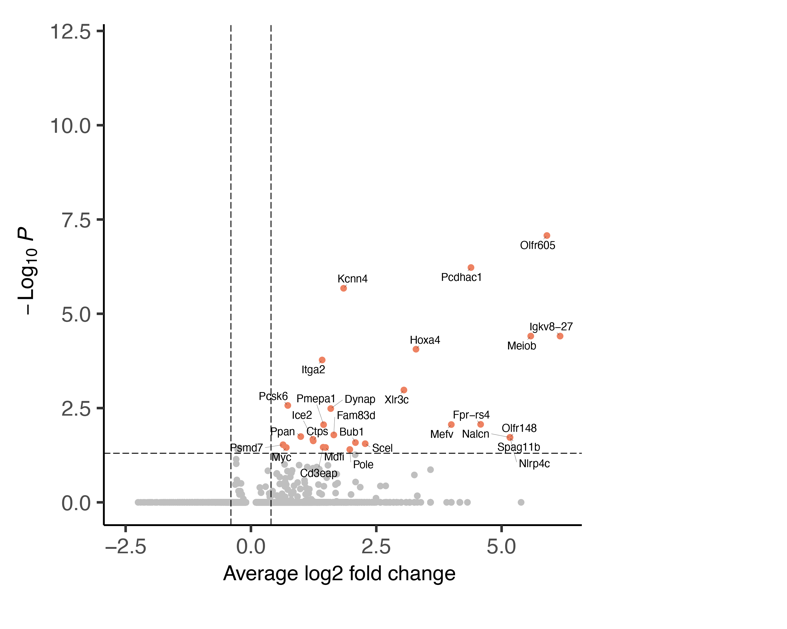


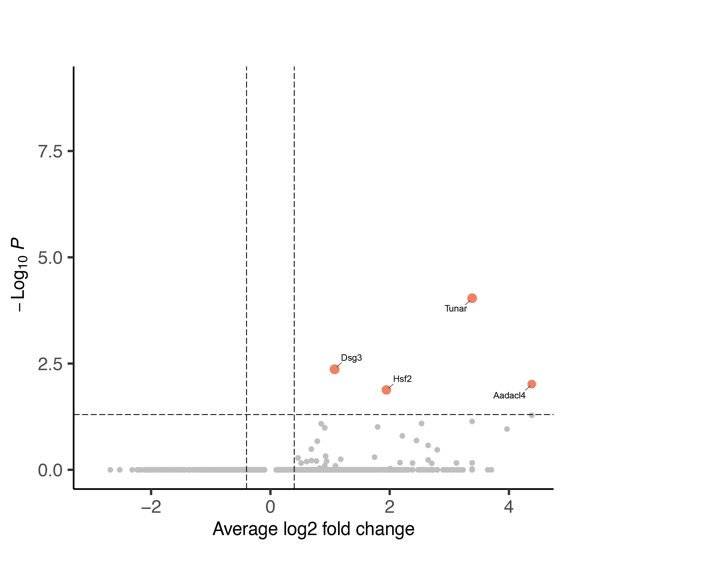

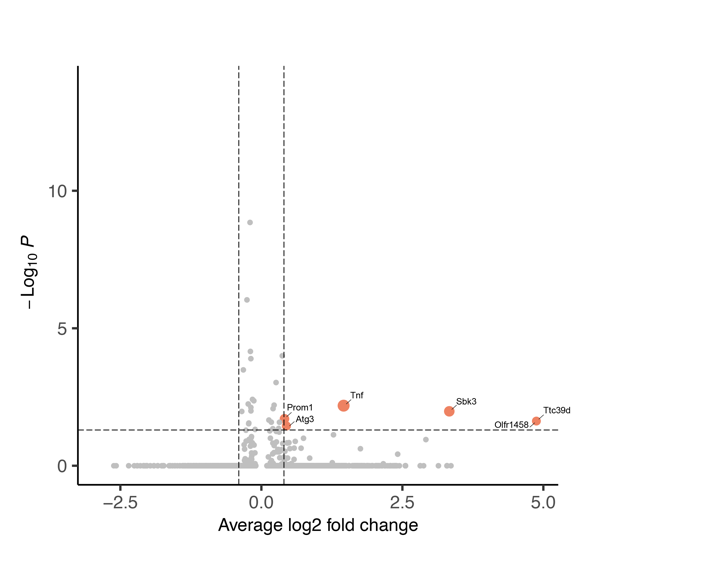


**Figure S8: Colocalization analysis.** Volcano plots of the differentially expressed (DE) host genes in Salmo^+^ spots of the *GsdmD*^-/-^ infected condition (left), the *Nlrc4*^-/-^ infected condition (middle), and the WT infected condition (right). In orange: significant DE genes.
