# Supplementary material for: ProbeST: a custom probe design pipeline for dual host–pathogen Spatial Transcriptomics
Source: BMC Genomics. 2026 Jun 25;27:561. doi: 10.1186/s12864-026-13077-z (PMC13295851; doi:10.1186/s12864-026-13077-z)
Supplement: Supplementary file 7 — Supplementary Material 7. [file 12864_2026_13077_MOESM7_ESM.pdf]

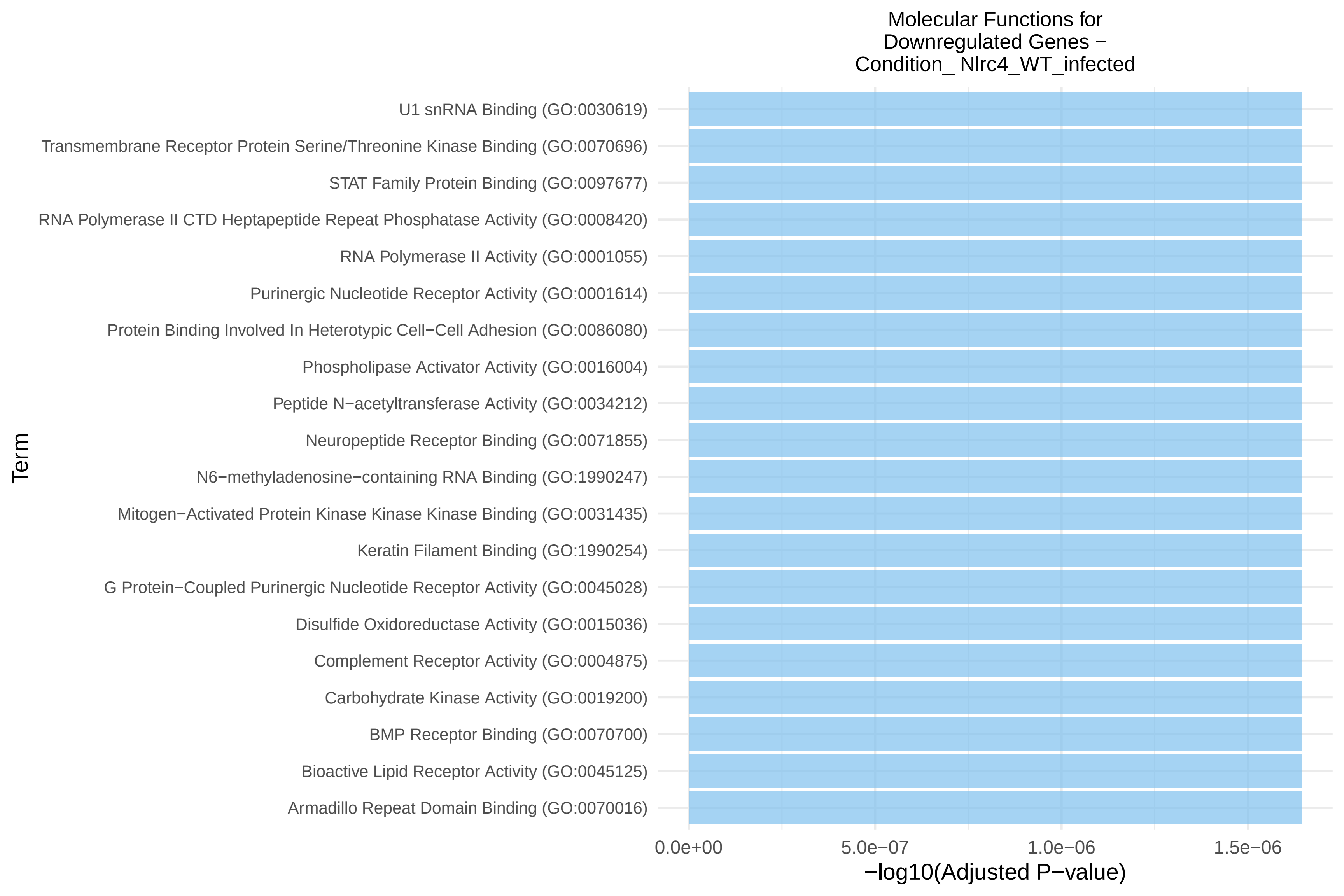
Molecular Functions for
Downregulated Genes −
Condition_ Nlrc4_WT_infected
Term
U1 snRNA Binding (GO:0030619)
Transmembrane Receptor Protein Serine/Threonine Kinase Binding (GO:0070696)
STAT Family Protein Binding (GO:0097677)
RNA Polymerase II CTD Heptapeptide Repeat Phosphatase Activity (GO:0008420)
RNA Polymerase II Activity (GO:0001055)
Purinergic Nucleotide Receptor Activity (GO:0001614)
Protein Binding Involved In Heterotypic Cell−Cell Adhesion (GO:0086080)
Phospholipase Activator Activity (GO:0016004)
Peptide N−acetyltransferase Activity (GO:0034212)
Neuropeptide Receptor Binding (GO:0071855)
N6−methyladenosine−containing RNA Binding (GO:1990247)
Mitogen−Activated Protein Kinase Kinase Kinase Binding (GO:0031435)
Keratin Filament Binding (GO:1990254)
G Protein−Coupled Purinergic Nucleotide Receptor Activity (GO:0045028)
Disulfide Oxidoreductase Activity (GO:0015036)
Complement Receptor Activity (GO:0004875)
Carbohydrate Kinase Activity (GO:0019200)
BMP Receptor Binding (GO:0070700)
Bioactive Lipid Receptor Activity (GO:0045125)
Armadillo Repeat Domain Binding (GO:0070016)
0.0e+00
5.0e−07
1.0e−06
1.5e−06
−log10(Adjusted P−value)
