# Supplementary figures and images for: ProbeST: a custom probe design pipeline for dual host–pathogen Spatial Transcriptomics
Source: BMC Genomics. 2026 Jun 25;27:561. doi: 10.1186/s12864-026-13077-z (PMC13295851; doi:10.1186/s12864-026-13077-z)

GO Biological Processes for  
Upregulated Genes – Condition  
WT\_infected

Term

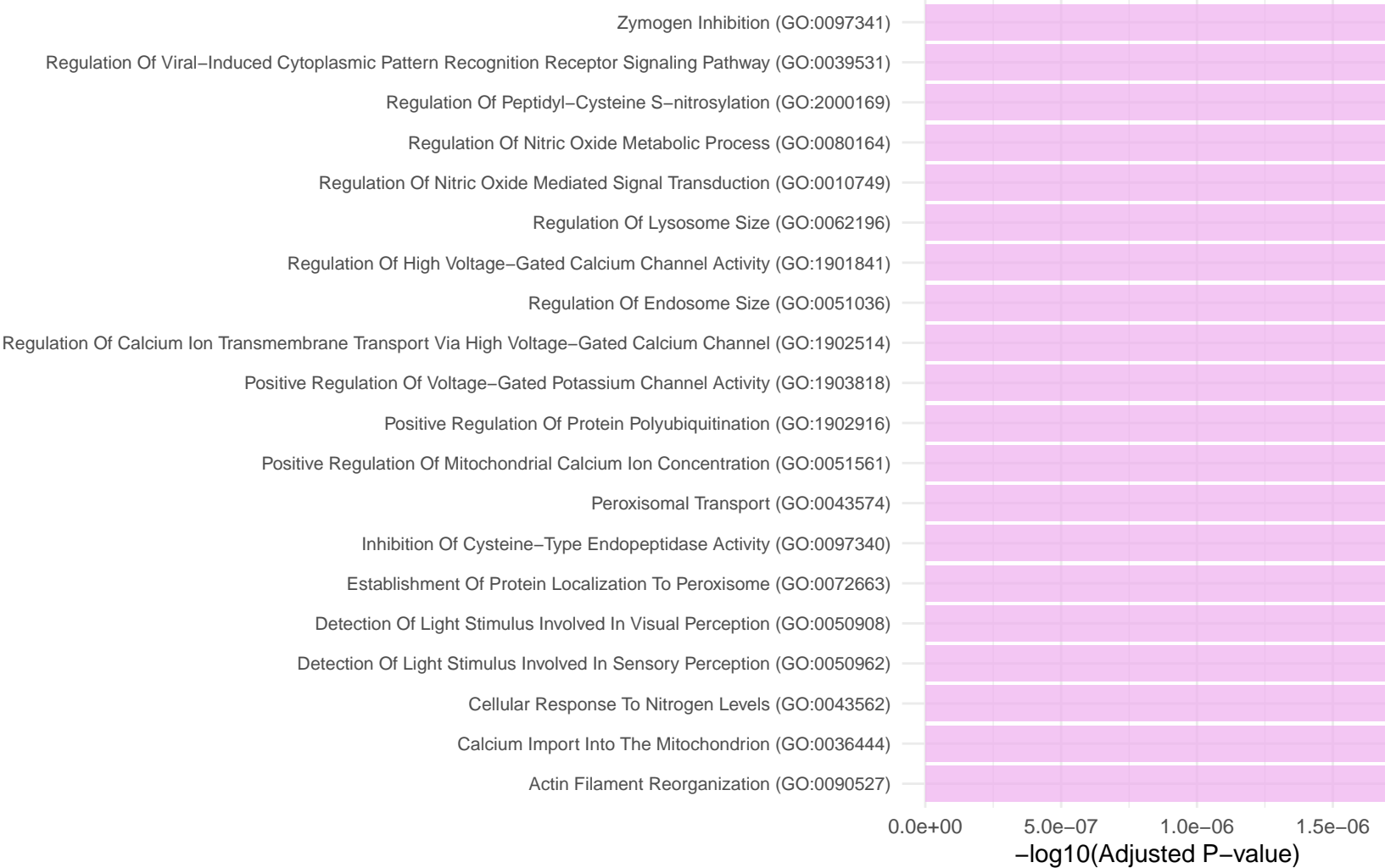

Supplement: Supplementary file 8 — Supplementary Material 8. [file 12864_2026_13077_MOESM8_ESM.pdf]
